# Effects of Different Lignin Contents and Water Contents on the Performance of DES-Based Hydrogels

**DOI:** 10.3390/gels12080710

**Published:** 2026-08-11

**Authors:** Panrong Guo, Xiaobo Xue, Mengxin Liu, Yunming Zou, Xian Wang, Jiongjiong Li, Fei Xiao, Xiangmeng Chen, Cheng Li, Hanyin Li, Zhongjian Li

**Affiliations:** 1 College of Horticulture, Henan Agricultural University, Zhengzhou 450026, China; 2 College of Forestry, Henan Agricultural University, Zhengzhou 450026, China; 3 College of Materials Science and Technology, Nanjing Forestry University, Nanjing 210037, China; 4 Hunan Academy of Forestry, Changsha 410018, China; 5 College of Pharmacy, Changsha Medical University, Changsha 410219, China

**Keywords:** water content, lignin, DES gel, deep eutectic solvent

## Abstract

This study fabricated choline–acrylic acid deep eutectic solvent (DES) hydrogels via in situ free-radical polymerization and systematically investigated the individual and co-optimization effects of lignin dosage and water content on the chemical structure, micromorphology, compressive mechanical properties, swelling behavior, and thermal stability of the hydrogels. This work quantitatively uncovers the co-optimization mechanism between the two variables in modulating crosslink density and pore architecture, thereby filling a research gap in the dual-factor co-optimization of biomass-based DES hydrogels. The results reveal that a moderate lignin dosage (0.02 g) generates abundant dynamic hydrogen bonds, densifying the crosslinked network and raising the maximum compressive stress from 0.378 MPa to 0.426 MPa, whereas excessive lignin triggers molecular aggregation and deteriorates mechanical performance. Higher water content dilutes crosslinking sites, reduces network compactness, boosts the swelling ratio while lowering compressive strength, and exerts negligible impacts on thermal degradation characteristics. FTIR analysis confirms that lignin participates in network formation solely through non-covalent hydrogen bonds, without forming new covalent bonds. A comprehensive performance evaluation identifies the optimal formulation as 0.02 g lignin and 60 g water. Although this two-factor optimization strategy provides clear experimental and theoretical guidance for designing sustainable soft materials, the present work still has limitations, including the use of only static laboratory characterizations, with no cyclic mechanical measurements or aging assessments. This study advances the customized performance tuning of lignin-derived DES hydrogels and facilitates the high-value valorization of lignin, which is promising for multifunctional green-material applications, including adsorption, flexible electronics, and biological carriers.

## 1. Introduction

Hydrogels, as a typical class of soft materials with three-dimensionally crosslinked macromolecular network structures [1], exhibit broad application prospects [2] in biomedicine, flexible electronics, environmental adsorption [3], intelligent actuation, and agricultural forestry processing owing to their high water content, excellent swelling behavior, favorable viscoelasticity, tunable mechanical properties, and biocompatibility [4,5]. Traditional hydrogels are usually fabricated in aqueous systems, where hydrophilic polymer networks are formed through chemical crosslinking or physical entanglement. They can absorb dozens to thousands of times their own mass of water within a short time while maintaining structural stability, with certain deformability and recoverability [6]. However, conventional hydrogel systems generally suffer from low mechanical strength, insufficient structural stability, poor environmental resistance, and insufficient multifunctionality [7]. Under harsh conditions such as high load, extreme humidity, elevated temperature, or complex chemical environments, they are prone to excessive swelling, structural collapse, mechanical failure, and rapid degradation [8,9,10,11], which restricts their applications as high-performance structural materials and long-term functional devices [12]. Therefore, developing novel hydrogel materials with enhanced mechanical robustness, structural stability, and multifunctional integration through green solvent engineering, biomass modification, and microstructure regulation has become an important research direction in soft materials science.

Deep Eutectic Solvent (DES) is a low-melting-point eutectic mixture formed by intermolecular hydrogen bonding interactions between hydrogen bond acceptors (HBAs) and hydrogen bond donors (HBDs) [13]. Similar to ionic liquids, DESs exhibit excellent physicochemical properties and offer additional advantages, such as low cost, facile preparation, good biocompatibility, and environmental friendliness [14]. More importantly, DES systems feature dense, dynamic hydrogen-bonding networks that provide stable, mild reaction environments for monomer polymerization, macromolecular crosslinking, and inorganic/organic recombination [15,16,17]. Compared with traditional aqueous solution systems, DES can serve not only as efficient solvents but also as structural participants in gel network formation, regulating polymer chains through hydrogen bonding, electrostatic interactions, and hydrophobic interactions [18]. As a result, DES-based gels generally exhibit improved mechanical strength, structural stability, and environmental adaptability [19,20,21]. In particular, the choline chloride (ChCl)–acrylic acid (AA) DES system features abundant active sites, including hydroxyl and carboxyl groups, and exhibits excellent compatibility with acrylic monomers, making it a promising medium for in situ free-radical polymerization and high-performance gel fabrication [22].

Lignin is a renewable aromatic polymer with reserves second only to those of cellulose. As a high-yield byproduct of forestry and papermaking industries, it features abundant sources, low cost, and high carbon content [23,24]. Structurally, lignin macromolecules are composed of phenylpropane units linked by C–C bonds and ether bonds, with numerous reactive functional groups such as phenolic hydroxyl, aliphatic hydroxyl, carboxyl, methoxy, and carbonyl groups [25]. These functional groups enable strong interactions with metal ions, polar polymers, and inorganic fillers, thereby imparting significant potential in reinforcement, adsorption, and network regulation [26,27,28,29]. Incorporating alkali lignin into gel systems not only enables the high-value utilization of lignin waste but also enhances mechanical properties, thermal stability, and functional performance through its rigid aromatic structure and multiple active sites [30,31]. Meanwhile, the introduction of lignin can improve the sustainability of gel materials without significantly compromising biocompatibility, which aligns with the development trends of green chemistry and low-carbon materials [32]. Therefore, combining lignin with DES gels offers a promising route to construct sustainable biomass-modified DES gels with enhanced structural and functional properties.

Recent studies have demonstrated the feasibility of incorporating lignin into DES-based gel systems [33]. Cheng et al. [34] prepared quaternized lignin–cellulose nanofiber (QALCNF)-based hydrogels with different lignin contents from bagasse using a deep eutectic solvent (DES). They found that lignin can regulate the pore structure of hydrogels through quinone/catechol groups, significantly improving their adhesion and mechanical properties. The optimal sample exhibited high toughness, adhesive strength, porosity, drug release, antibacterial activity, and cytocompatibility. This study provides a theoretical basis for the design and development of high-performance lignin–cellulose nanofiber-based bio-hydrogels. Yang et al. [30] prepared a polymerizable DES/lignin composite hydrogel with high transparency, UV resistance, adhesion, conductivity, self-healing ability, and wide-temperature environmental stability. In addition, lignin-derived components have been used to produce conductive and functional gels for sensing, environmental monitoring, and flexible device applications. Zhang et al. [35] used a glycerol–choline chloride-based DES and investigated the physicochemical properties of DES–water mixtures with different water contents and their effects on poplar pretreatment. The results showed that adding 10–15% water promoted lignin dissolution and extraction efficiency, while 35% water reduced DES viscosity by 95%. After pretreatment, the solid cellulose purity exceeded 80% and the enzymatic hydrolysis rate reached over 95%. Meanwhile, lignin purity, active hydroxyl content, and molecular weight distribution were significantly improved. This study provides a feasible route to industrialize DES-based lignocellulose pretreatment and preferential lignin extraction. Du et al. [36] prepared poly(methacryloyloxyethyltrimethylammonium chloride)-glycerol (PDMC-Gly) gels using hemicellulose–lignin complexes (HLC) extracted from rice straw as physical crosslinkers. After adding 2 wt% HLC, the tensile strength (846.2 kPa) and elastic modulus (290.0 kPa) of the gels were greatly enhanced. The gels also showed excellent light transmittance and UV-blocking performance, with an ionic conductivity of 1.61 × 10^3^ mS/cm. These gels have potential applications in human motion detection, environmental monitoring, and fire warning, offering new insights into the development of high-performance, flexible, conductive gels. However, systematic studies on the synergistic regulation of gel structure and properties by lignin addition and system water content remain scarce. Lignin dosage directly affects crosslink density, network homogeneity, pore structure, and mechanical performance of gels. At low dosage, lignin provides limited reinforcement, whereas excessive lignin may induce agglomeration, phase separation, and over-crosslinking, increasing brittleness and reducing mechanical strength [37]. Similarity, water content strongly affects the morphology, microstructure, swelling behavior, and mechanical properties. Insufficient water can lead to high viscosity, uneven monomer diffusion, and incomplete polymerization, whereas excessive water may dilute crosslinking sites, lower network density [38], and result in a loose structure with low strength and excessive swelling. In practical applications, the key characteristics and functions of DES gels are determined by mechanical properties, swelling behavior, microstructure, and thermal stability, all of which strongly depend on the synergistic control of lignin dosage and water content.

Based on these scientific challenges, lignin-based deep eutectic solvent (DES) gels were fabricated in this study via in situ free radical polymerization using choline chloride (ChCl)–acrylic acid (AA) DES as the reaction medium and functional component, industrial alkali lignin (AL) as the biomass modifier, N,N’-methylenebisacrylamide (MBA) as the crosslinker, and ammonium persulfate (APS) as the initiator. Although numerous previous studies have explored the separate effects of lignin dosage or water content on gel performance [39,40,41,42], few investigations systematically uncover the co-optimization coupling mechanism through which lignin loading and water content jointly modulate the crosslink density, pore microstructure, mechanical strength, swelling behavior, and thermal stability of DES gels. This critical research gap restricts the precise performance tuning of biomass-derived DES gels. By varying lignin dosage and water content, the present work systematically evaluates the comprehensive influences of preparation conditions on the chemical structure, micromorphology, compressive mechanical properties, swelling performance, and thermal stability of DES gels. Furthermore, the interaction mechanism of lignin within the DES gel matrix and the regulatory mechanism of water content during network formation are elucidated, and the optimal preparation parameters are identified. This study partially fills the existing research gap on the dual-factor co-optimization regulation of lignin-modified DES gels, providing experimental evidence and theoretical guidance for the design, fabrication, and application of high-performance biomass-based DES gels. It facilitates the high-value valorization of lignin and advances the development of sustainable, multifunctional soft materials for adsorption separation, flexible electronics, biological carriers, adhesives, and other fields.

## 2. Results and Discussion

### 2.1. Designation and Composition of DES Gels

In the experiment, lignin dosages were set at 0, 0.02, 0.06, 0.1, 0.2, and 0.4 g, with a fixed water content of 60 g, and the as-prepared DES gels were denoted as Gel-L1-W2, Gel-L2-W2, Gel-L3-W2, Gel-L4-W2, Gel-L5-W2, and Gel-L6-W2, respectively. Meanwhile, DES gels with a fixed lignin dosage of 0.02 g and varying water contents of 40, 60, and 80 g were prepared and named Gel-L2-W1, Gel-L2-W2, and Gel-L2-W3, respectively (Table 1).

**Table 1:** Composition and Naming of DES Hydrogel Samples.

Sample Code	ChCl/g	AA/g	MBA/g	APS/g	AL/g	H_2_O/g
Gel-L1-W2	9.84	10.16	0.12	0.12	0	60
Gel-L2-W2	9.84	10.16	0.12	0.12	0.02	60
Gel-L3-W2	9.84	10.16	0.12	0.12	0.06	60
Gel-L4-W2	9.84	10.16	0.12	0.12	0.1	60
Gel-L5-W2	9.84	10.16	0.12	0.12	0.2	60
Gel-L6-W2	9.84	10.16	0.12	0.12	0.4	60
Gel-L2-W1	9.84	10.16	0.12	0.12	0.02	40
Gel-L2-W2	9.84	10.16	0.12	0.12	0.02	60
Gel-L2-W3	9.84	10.16	0.12	0.12	0.02	80

### 2.2. FTIR Analysis of DES Gels

**Figure 1:**
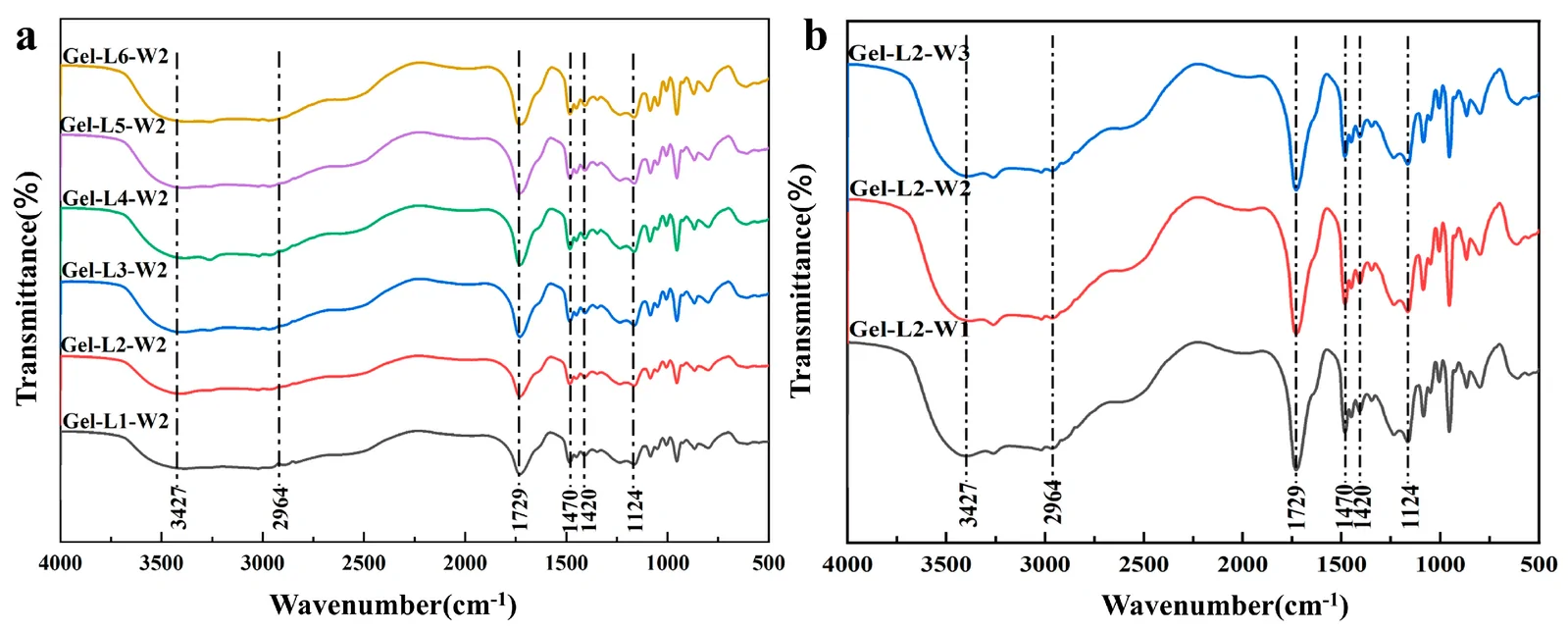
FT-IR spectra of hydrogels with different lignin additions (**a**) and water contents (**b**).

Figure 1 shows the Fourier-transform infrared (FT-IR) spectra of DES gels prepared under different lignin loadings and water contents. The broad absorption peak at 3427 cm^−1^ is attributed to the stretching vibration of aromatic and phenolic hydroxyl groups (-OH), originating from both the DES matrix and the incorporated lignin component [43]. The characteristic peak at 1729 cm^−1^ corresponds to the stretching vibration of carbonyl groups (C=O) in the acrylic polymer network [44]. The peaks at 2964 cm^−1^ and 1470 cm^−1^ are assigned to the stretching and bending vibrations of methylene groups (-CH_2_-) [45], respectively, which are typical characteristic peaks of the DES-based polymer backbone. The absorption peak near 1124 cm^−1^ arises from the C-O stretching and deformation vibrations of methylene ether linkages in the cross-linked network [46]. Upon the incorporation of lignin, the intensity of the absorption band around 1420 cm^−1^ increased. This enhancement, superimposed on the intrinsic C-H bending vibration of the poly(acrylic acid) matrix [47,48], confirms the successful integration of lignin, whose aromatic skeletal vibrations typically overlap in this region. Meanwhile, comparison of the FT-IR spectra of all gradient samples reveals that the positions of characteristic peaks of the gel samples, regulated by different lignin loadings and water contents, are highly consistent, with no new functional-group characteristic peaks appearing and no original core characteristic peaks disappearing or shifting significantly [49]. These results indicate that lignin mainly participates in the formation of the gel network through non-covalent interactions, such as hydrogen bonds, without forming new covalent bonds, and that water serves only as a dispersant during polymerization. Both factors merely regulate the degree of physical cross-linking and the microscopic pore structure of the gel, without altering the basic chemical structure or core cross-linking mechanism of the DES gel matrix, thereby fully demonstrating that this biomass-modified DES gel system exhibits excellent chemical structural stability.

It should be pointed out that there is no obvious characteristic peak of lignin that is obviously separated from the DES gel matrix in the Fourier transform infrared spectrum, which is mainly due to the obvious peak overlap between the aromatic skeleton vibration of lignin and the poly (acrylic acid) skeleton, and the relatively low lignin content (≤0.4 g) compared with the DES matrix. However, successful incorporation of lignin can be demonstrated by an enhancement in absorption at around 1420 cm^−1^, which directly reflects the additional hydroxyl groups contributed by lignin. These spectral changes, together with the dose-dependent changes in the gel properties (discussed in Section 2.4, Section 2.5 and Section 2.6), jointly confirmed that lignin is integrated into the gel network at the molecular level without forming a separate phase domain.

### 2.3. Microstructural Analysis of DES Gels

**Figure 2:**
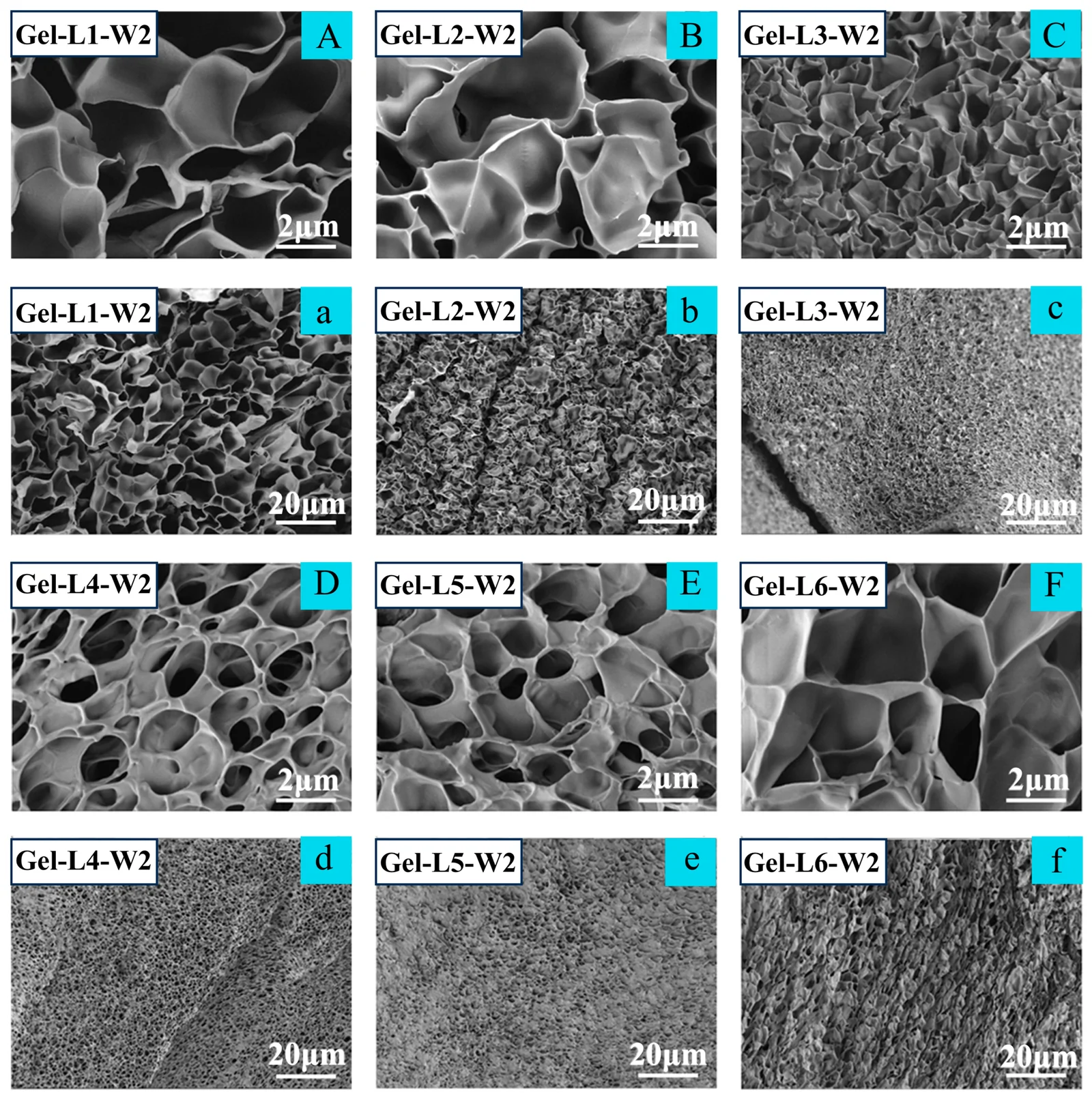
SEM images of DES gels with different lignin loadings: (**A**) Gel-L1-W2, 2 μm (**B**) Gel-L2-W2, 2 μm; (**C**) Gel-L3-W2, 2 μm; (**a**) Gel-L1-W2, 20 μm; (**b**) Gel-L2-W2, 20 μm; (**c**) Gel-L3-W2, 20 μm; (**D**) Gel-L4-W2, 2 μm; (**E**) Gel-L5-W2, 2 μm; (**F**) Gel-L6-W2, 2 μm; (**d**) Gel-L4-W2, 20 μm; (**e**) Gel-L5-W2, 20 μm; (**f**) Gel-L6-W2, 20 μm.

**Figure 3:**
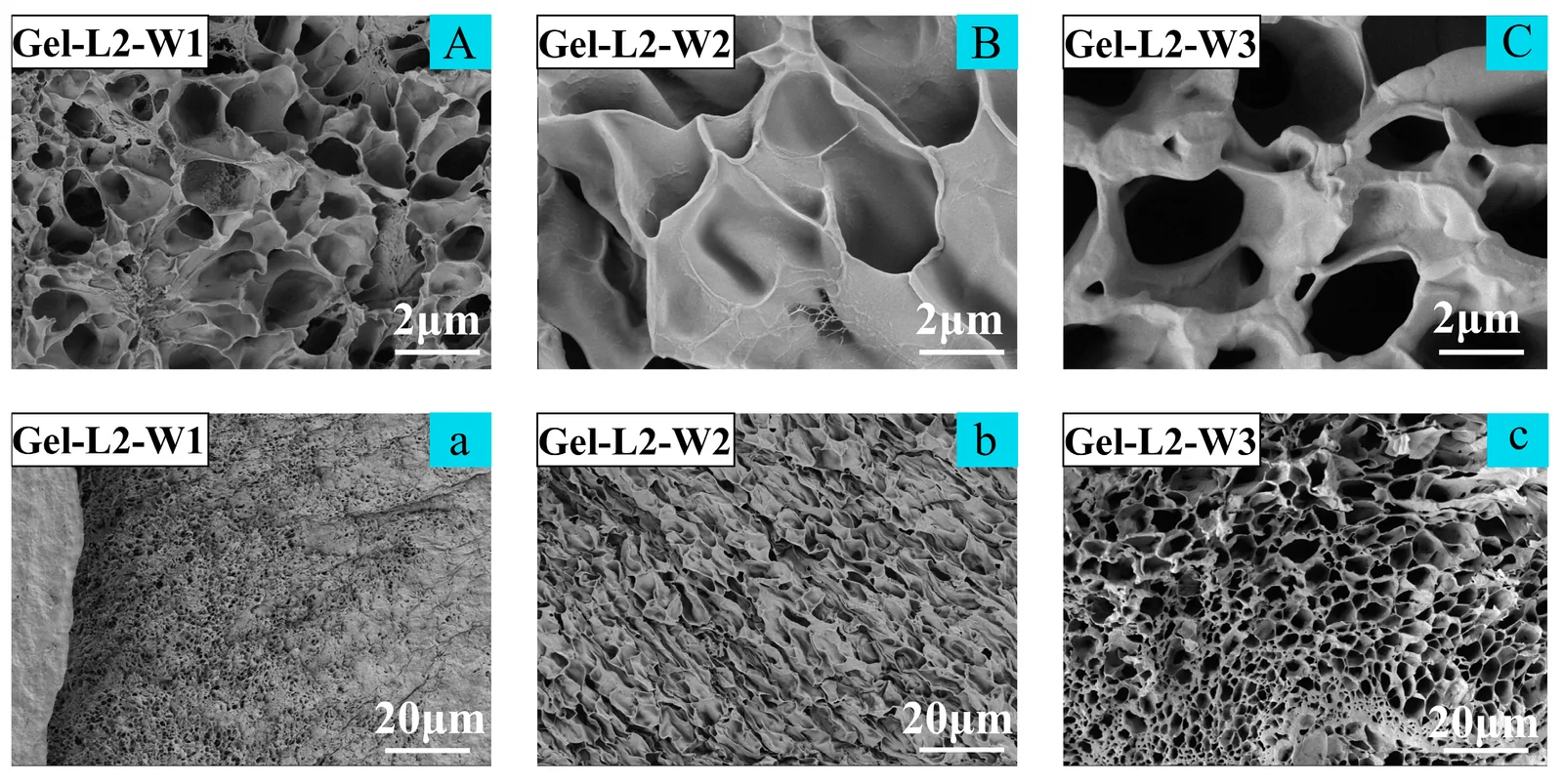
SEM images of DES gels with different water contents: (**A**) Gel-L2-W1, 2 μm (**B**) Gel-L2-W2, 2 μm; (**C**) Gel-L2-W3, 2 μm; (**a**) Gel-L2-W1, 20 μm; (**b**) Gel-L2-W2, 20 μm; (**c**) Gel-L2-W3, 20 μm.

Figure 2 and Figure 3 show the cross-sectional scanning electron microscopy (SEM) images of DES gels prepared with different lignin loadings and different water contents, respectively. All freeze-dried DES gel samples exhibit a typical honeycomb-like interconnected porous structure [50]. The formation of such a network porosity originates from the freeze-drying mechanism of the gels: after water absorption and swelling, the internal water of the gel solidifies into ice crystals at an ultra-low temperature, and the ice crystals are directly removed by sublimation during freeze-drying, thus perfectly preserving the original porous network structure of the gel [51].

As shown in Figure 2, compared with the blank sample Gel-L1-W2 without lignin, the pore structure of the DES gel becomes notably denser and more uniform with increasing lignin loading. This phenomenon is mainly determined by the structural characteristics of lignin [52,53,54,55]. Lignin possesses a complex three-dimensional conformation with numerous active groups (e.g., hydroxyl and carboxyl groups) distributed along its molecular backbone, which can form abundant hydrogen bonds with the polymer chains of the DES gel matrix [56]. The physical cross-linking effect of hydrogen bonds tightens the gel network and enhances its structural compactness. As lignin loading increases, the number of cross-linking sites continues to rise, and the network becomes increasingly dense. However, an excessively high lignin content can lead to local agglomeration, resulting in a slight decrease in network uniformity.

As shown in Figure 3, varying the water content does not alter the overall honeycomb-like porous morphology of the gel, and the pore structure remains unchanged. Nevertheless, the gel network becomes progressively looser as water is added. This is because water can dilute the cross-linking sites and reduce the network compactness during the gel-forming polymerization process [57,58]. These results indicate that water content is a key factor for regulating the network density of DES gels. The tunable network structure can be achieved by adjusting the water content to meet the requirements of diverse applications such as adsorption separation, mass transport, and carrier loading.

One notable observation from the SEM analysis is that lignin is not a distinct particle or a separated structural domain in any microscopic photograph. This is due to the amorphous nature of lignin and its tight molecular-level integration with the polymer matrix, resulting in relatively poor performance under standard SEM imaging conditions. The absence of visible lignin particles does not mean the absence of lignin incorporation; instead, it supports the findings of Fourier transform infrared spectroscopy (Section 2.2), namely that lignin is well-dispersed and participates in network formation through non-covalent hydrogen bonds without forming separate phase domains. With changes in lignin dosage, the systematic and dose-dependent changes in swelling behavior (Section 2.4), thermal stability (Section 2.5), and mechanical properties (Section 2.6) confirm that lignin has been successfully and uniformly incorporated into the DES gel network.

### 2.4. Swelling Performance Analysis of DES Gels

**Figure 4:**
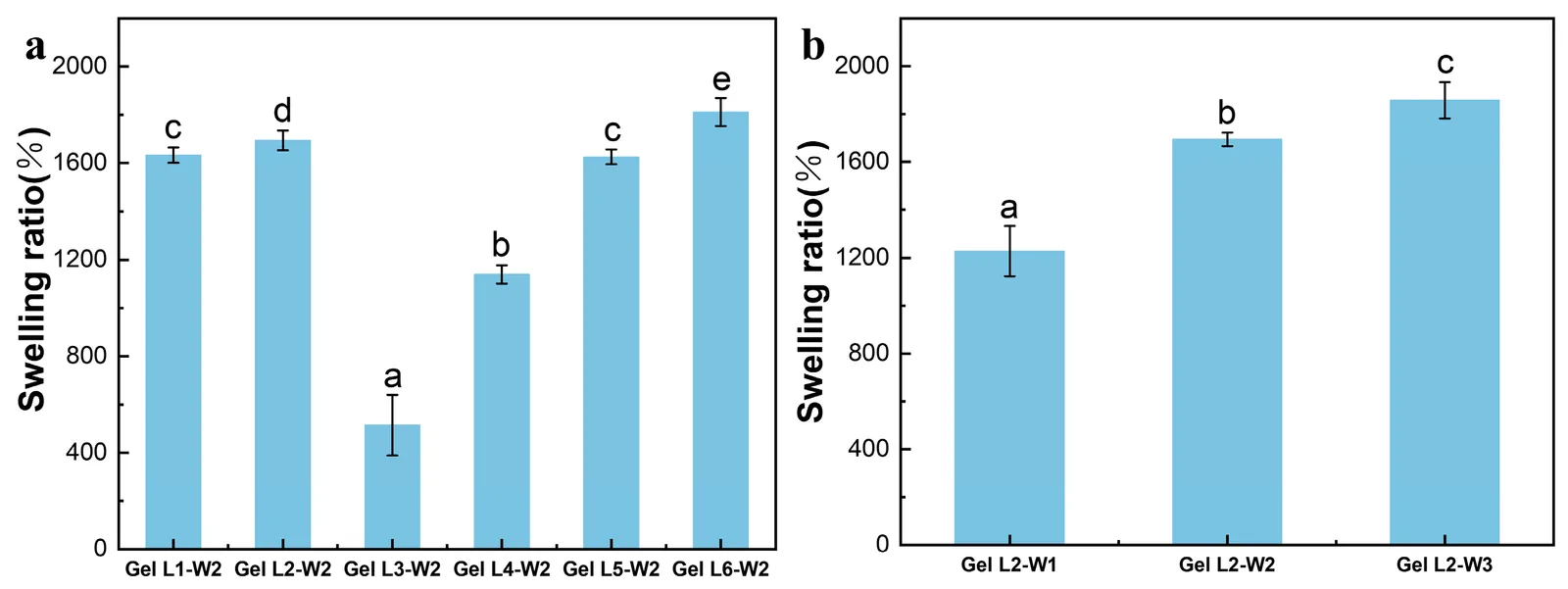
Swelling curves of DES gels with different lignin loadings (**a**) and water contents (**b**). Note: Data are presented as mean ± SD (*n* = 3); different lowercase letters indicate significant differences (*p* < 0.05).

Figure 4 presents the swelling profiles of DES gels with varying lignin dosages and water contents. Water absorption and swelling are characteristic properties of hydrogels, and the DES gels exhibit a favorable water-swelling capacity owing to their porous three-dimensional network architectures. The figures show that the swelling behavior of the gels exhibits a non-monotonic trend with increasing lignin loading in the gel matrix [59]. At low lignin concentrations (Gel L3-W2), the swelling ratio decreases remarkably. This decline can be attributed to the incorporation of lignin as physical crosslinking sites; abundant hydroxyl groups on lignin form strong hydrogen bonds with the polymer matrix, generating a denser network structure that restricts chain segment mobility and water uptake. With further elevation of lignin dosage, the swelling ratio rebounds and even surpasses that of the control group, indicating a structural transformation. Excess lignin may enlarge the free volume within the network or introduce additional hydrophilic binding sites. The gel with a lignin loading of 0.4 g displays a slightly higher swelling ratio than the lignin-free control gel. During water swelling, the interwoven internal network provides DES gels with abundant large pores, facilitating the diffusion of water molecules throughout the network [60]. After the pores are saturated with water, the polymer chains gradually extend, thereby completing the water-swelling process of the DES gels. Additionally, the swelling ratio of DES gels increases with increasing water content in the precursor system. The elevated water content during gel fabrication may enlarge the internal pore size of the resultant DES gels [61,62], enabling the gels to accommodate more water molecules and achieve a higher equilibrium swelling ratio.

### 2.5. Thermal Stability Analysis of DES Gels

**Figure 5:**
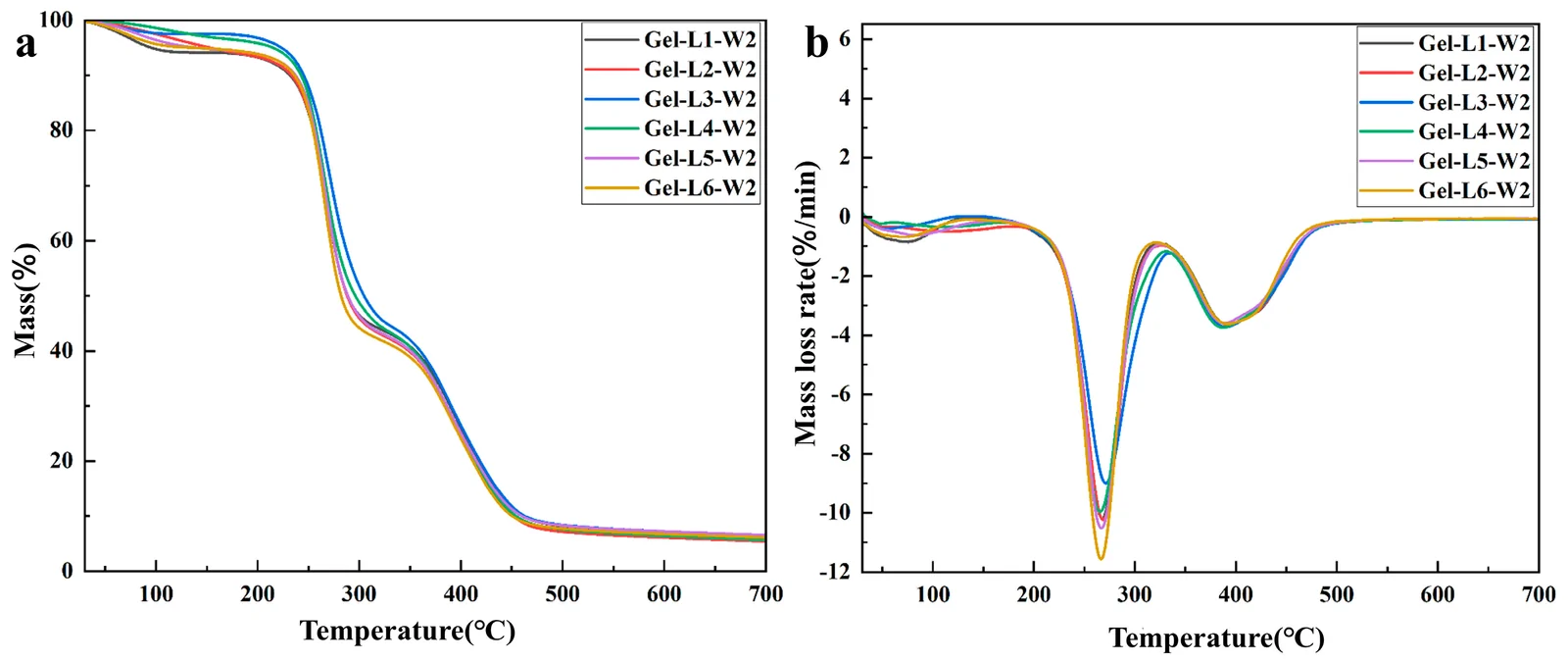
Thermogravimetric loss (**a**) and rate curves (**b**) of DES gels with different lignin loadings.

**Figure 6:**
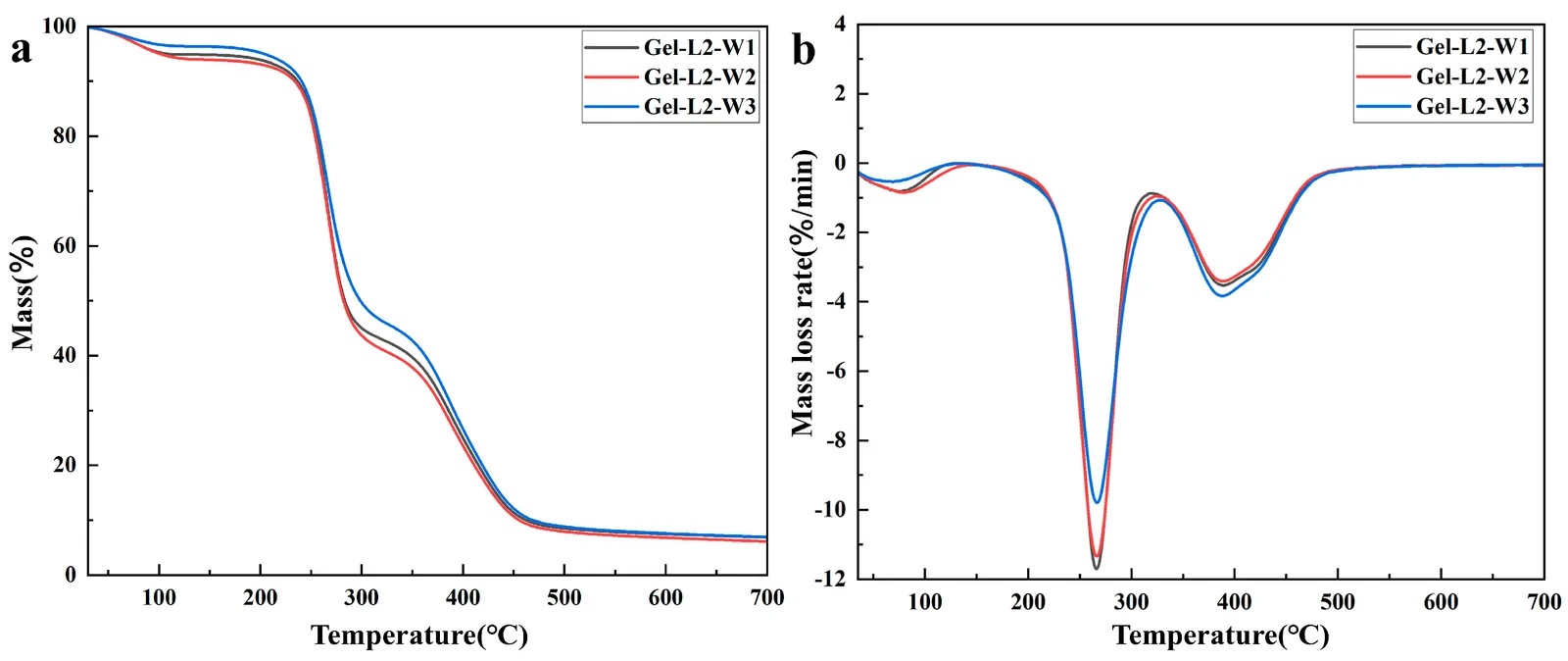
Thermogravimetric loss (**a**) and rate curves (**b**) of DES gels with different water contents.

Figure 5 and Figure 6 display the thermogravimetric loss and mass loss rate curves of DES gels with different lignin dosages and water contents, respectively, and Table 2 and Table 3 summarize the characteristic thermogravimetric parameters of all samples. As observed from the TGA and DTG curves of the hydrogels, the thermal degradation of DES gels can be generally divided into three stages. Stage I: Approximately 30–250 °C, with a slight mass loss of around 8%, which can be attributed to the volatilization of bound water inside the gels. Combined with the *T*_5%_ data in Table 2 and Table 3, adjusting the lignin dosage and water content slightly alters the temperature at which 5% mass loss is induced by water evaporation. The formulation with moderate lignin loading (Gel-L2-W2) and appropriate water content slightly increases the T5% value and alleviates the low-temperature mass loss due to water escape [63]. Stage II: Approximately 250–460 °C, where the gels undergo drastic and substantial mass loss. Two distinct mass-loss peaks are observed on the DTG curves, corresponding to *T_max_*_1_ and *T_max_*_2_. This phenomenon arises from the stepwise decomposition of the polymer matrix and lignin components into small molecules and gaseous products at temperatures within this range. Comparison of the tabulated parameters reveals that the peak temperatures of the two thermal degradation stages fluctuate little across all groups, and that only marginal differences exist in the maximum mass loss rates. The introduction of lignin and adjustment of water content merely slightly modify the degradation rate without shifting the overall thermal decomposition temperature range [64]. Stage III: Approximately 460–700 °C, accompanied by negligible mass reduction. This minor weight loss is attributed to incomplete carbonization of trace residual substances, whereas the majority of thermal degradation residues have been converted into carbonaceous char [65,66]. Combining the curve profiles and char residue data from the tables, all samples exhibit nearly identical thermal degradation procedures regardless of lignin dosage or water content. The char yields of all formulations at 700 °C range narrowly from 8.5% to 9.1% with trivial deviations. These results demonstrate that increasing lignin loading or water content exerts a negligible influence on the thermal stability of the as-prepared DES gels.

### 2.6. Mechanical Properties Analysis of DES Gels

**Figure 7:**
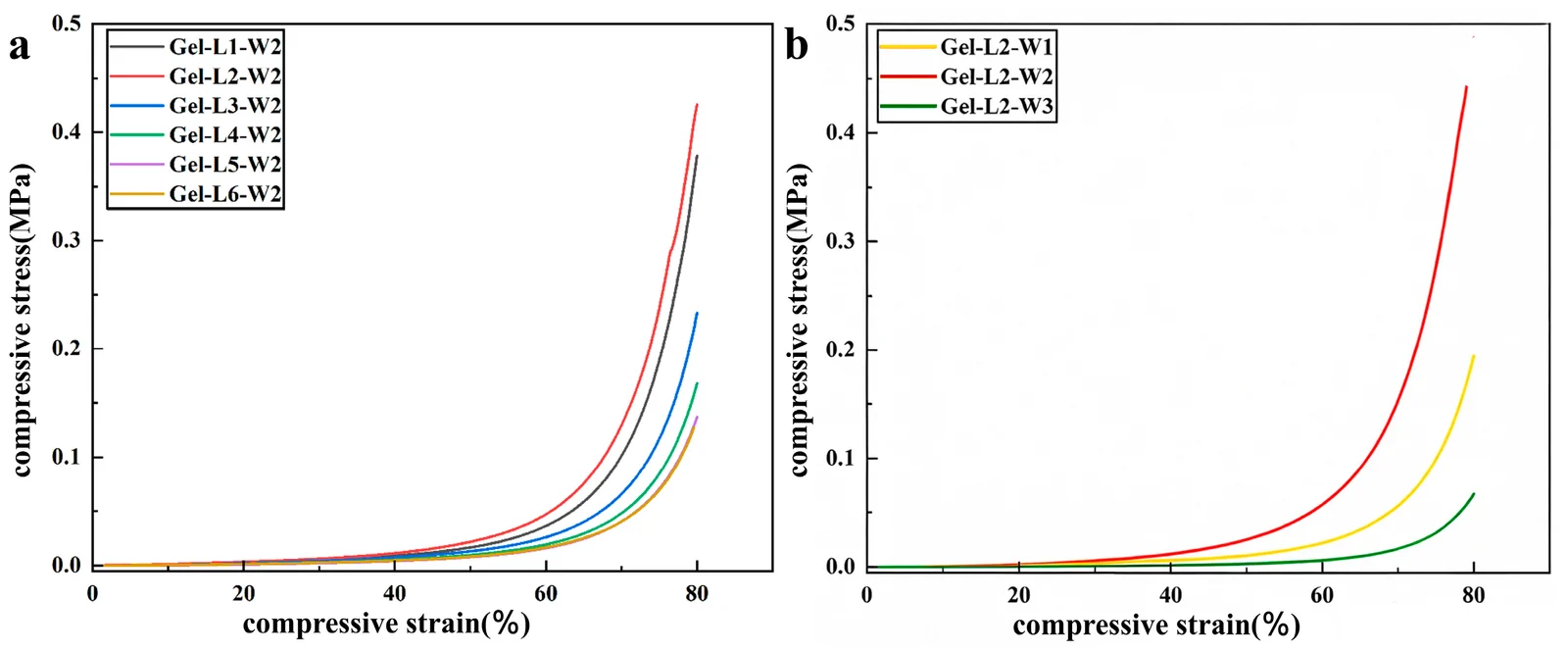
Compressive Stress–Strain Diagrams of DES Gels with Different Lignin Contents (**a**) and Water Contents (**b**).

**Figure 8:**
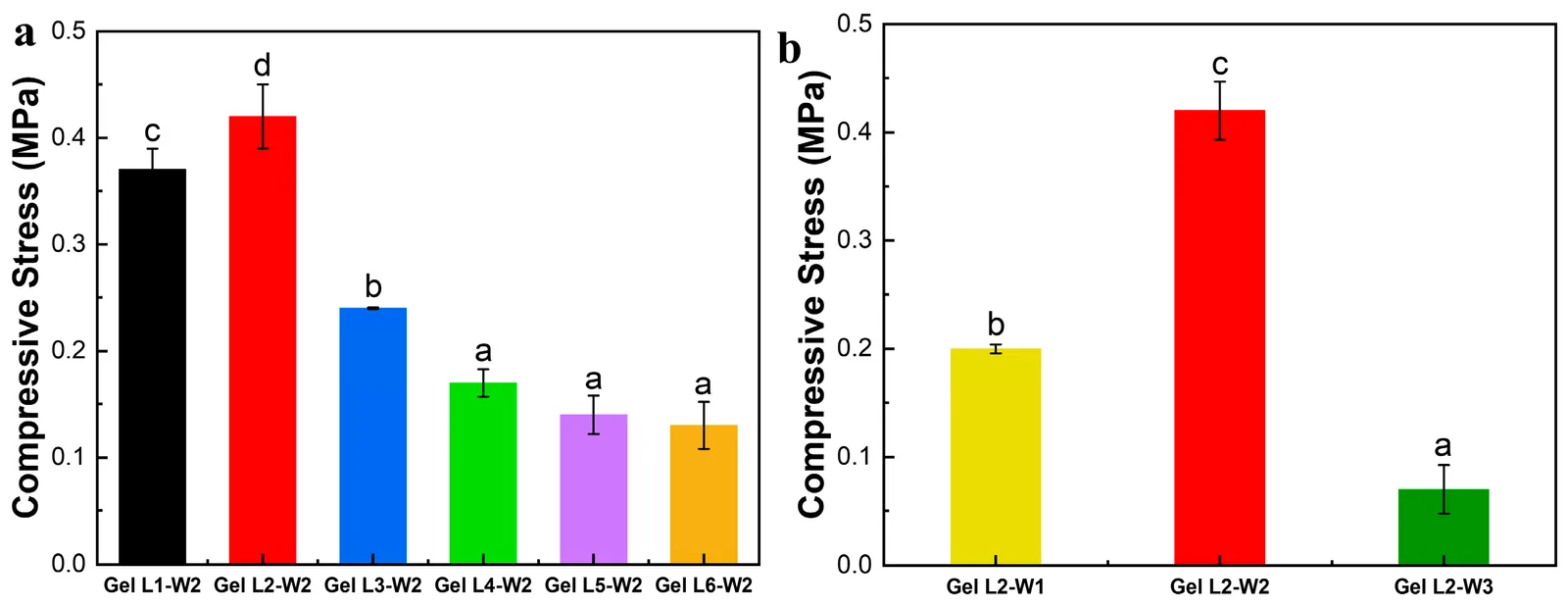
The maximum compressive stress of DES Gels Gels with Different Lignin Contents (**a**) and Water Contents (**b**). Note: Data are presented as mean ± SD (*n* = 3); different lowercase letters indicate significant differences (*p* < 0.05).

Figure 7 and Figure 8 present the compressive stress–strain curves of DES gels prepared with different lignin loadings and water contents. All gel samples exhibit consistent compressive mechanical behavior: compressive strain increases in step with increasing compressive stress. In the elastic deformation stage, where the compressive strain is below 50%, the three-dimensional porous network within the gel undergoes only reversible stretching and bending, leading to a gradual increase in compressive stress. Once the compressive strain exceeds 50%, the gel enters the densification stage, in which the interconnected internal pores are gradually compacted, and polymer chains stack tightly to bear external loads, leading to a rapid rise in compressive stress [67]. This behavior is typical of porous, soft materials under compression.

Figure 7 and Figure 8a illustrates the regulatory effect of lignin loading on the compressive performance of the gels. At a lignin loading of 0.02 g (Gel-L2-W2), the DES gel achieves a maximum compressive stress of 0.426 MPa, which is significantly higher than that of the neat gel without lignin (0.378 MPa, Gel-L1-W2). However, as lignin loading increases, the maximum compressive stress shows an overall decreasing trend. This phenomenon arises from the dual regulatory role of lignin in the cross-linked gel network: the abundant hydroxyl groups on the aromatic backbone of lignin can form numerous dynamic hydrogen bonds with the polymer chains of the DES matrix, acting as physical cross-linking points to strengthen the three-dimensional network and construct a uniform porous framework [68]. During compression, the covalently cross-linked network maintains the structural integrity of the gel, while the widely distributed hydrogen bonds undergo reversible cleavage and reformation to efficiently dissipate external compressive energy, thereby endowing Gel-L2-W2 with the optimal compressive performance. Excessive lignin tends to promote molecular aggregation and local over-crosslinking, disrupting the uniformity of the gel network and introducing internal structural defects [69,70], ultimately reducing the load-bearing capacity of the gel. Figure 7 and Figure 8b further reveal the regulatory effect of water content on the compressive behavior of hydrogels at the optimal lignin loading (0.02 g, L2). The compressive stress of the three groups follows the order of Gel-L2-W2 > Gel-L2-W1 > Gel-L2-W3. As the dispersion medium in the system, moderate water (W2) can fully disperse lignin and polymer chains, achieving uniform distribution of hydrogen-bond crosslinking sites and constructing a dense network, thereby endowing the hydrogel with outstanding compressive resistance [71]. When the water content decreases (W1), the solute concentration of the system increases, restricting the stacking of polymer and lignin chains and reducing the number of effective physical crosslinking sites, thereby weakening network rigidity and leading to inferior compressive performance compared with the W2 group [72]. Excessively high water content (W3) dilutes the crosslinking active sites, reduces the crosslinking density of the system, and enlarges the internal pore size, thereby weakening the supporting effect of the hydrogel skeleton; however, the flexibility and ductility of the hydrogel are effectively improved [73]. The co-optimization effect of these two parameters allows effective regulation of the mechanical properties of biomass-based DES gels.

## 3. Conclusions

In this study, choline chloride–acrylic acid DES hydrogels were successfully fabricated via in situ free-radical polymerization, and the independent and co-optimization effects of lignin dosage and water content on the network microstructure and core functional properties of the gels were systematically investigated. Key findings demonstrate that a moderate lignin dosage (0.02 g) introduces abundant dynamic hydrogen bonds, forming a denser cross-linked network and increasing the maximum compressive stress from 0.378 MPa to 0.426 MPa, whereas excessive lignin dosage induces molecular aggregation and local overcrosslinking, deteriorating mechanical performance. Higher water content dilutes cross-linking sites, reduces network compactness, and elevates the swelling ratio, thereby decreasing compressive strength, while exerting no obvious influence on thermal degradation behavior. Combined FTIR, SEM, and TGA characterizations verify that lignin participates in network formation solely through non-covalent hydrogen bonds, without altering the fundamental chemical skeleton of the DES matrix, and the optimal formulation, balancing overall performance, is identified as 0.02 g lignin paired with 60 g water. Unlike previous reports that only examined lignin or water content as a single variable, this work innovatively reveals the co-optimization mechanism between these two critical formulation parameters and quantitatively elucidates their co-regulatory rules for crosslink density, pore architecture, swelling capacity, and thermostability. This delivers a systematic two-factor optimization strategy that breaks the limitations of conventional single-variable research on biomass DES hydrogels, representing the core novelty of this work and providing direct experimental and theoretical guidance for designing high-performance biomass-derived soft materials. Despite the above achievements, several limitations remain in the present work. All characterizations involve only static laboratory tests, without dynamic cyclic mechanical measurements or long-term environmental aging assessments; furthermore, all property evaluations are conducted at room temperature, failing to simulate complex practical service environments with fluctuating temperatures and humidity. Because the alkali lignin was used directly without pretreatment, future industrial deployment will require the standardization of key raw-material properties, particularly the moisture content and particle size distribution of lignin. Such standardization will be essential for ensuring batch-to-batch consistency in the mechanical and swelling properties of the resulting gels. Future work will focus on cyclic compression, swelling–deswelling, and accelerated aging tests to evaluate the service durability of the hydrogels, alongside practical application validation, including heavy-metal adsorption, flexible sensing, and biological carrier performance under simulated operating conditions. The outcomes are expected to enable scalable industrial deployment of biomass DES hydrogels for environmental remediation, wearable electronics, and biomedicine delivery systems.

## 4. Materials and Methods

### 4.1. Experimental Materials

Choline chloride (ChCl, ≥98% purity, analytical grade), acrylic acid (AA, ≥99% purity, analytical grade), N,N’-methylenebisacrylamide (MBA, ≥99% purity, analytical grade), ammonium persulfate (APS, ≥98% purity, analytical grade), and alkali lignin (AL, BR) is a dark brown to black powder with a moisture content of 5.10%, a pH value of 9.77 (measured in a 5% aqueous solution), and a methoxy content of 10% were purchased from Shanghai Macklin Biochemical Technology Co., Ltd. (Shanghai, China). The lignin was not subjected to pretreatments such as drying to constant weight or sieving to a specific mesh size before use.

### 4.2. Experimental Methods

Mix chlorocitrate and acrylic acid in a 1:2 molar ratio, then heat and stir them in an 80 °C water bath until the mixture becomes a transparent liquid, obtaining the deep miscible solvent (DES) of chlorocitrate and acrylic acid. Accurately weigh different masses of industrial basic lignin (without pretreatment), and completely dissolve it in 20 g of DES through magnetic stirring. Next, stir to dissolve 0.12 g of the cross-linking agent N,N’-methylbisacrylamide (MBA) and 0.12 g of the initiator ammonium persulfate (APS), then dilute them with varying amounts of deionized water before homogenizing. Mix the DES solution containing lignin with the aqueous solutions of MBA and APS and stir evenly. Finally, heat and mechanically stir the mixture at 80 °C in a constant temperature water bath for 2 h to obtain the final gel sample [60]. In the experiment, lignin dosages were set at 0, 0.02, 0.06, 0.1, 0.2, and 0.4 g, with a fixed water content of 60 g, and the as-prepared DES gels were denoted as Gel-L1-W2, Gel-L2-W2, Gel-L3-W2, Gel-L4-W2, Gel-L5-W2, and Gel-L6-W2, respectively. Meanwhile, DES gels with a fixed lignin dosage of 0.02 g and varying water contents of 40, 60, and 80 g were prepared and named Gel-L2-W1, Gel-L2-W2, and Gel-L2-W3, respectively.

### 4.3. Structural and Performance Characterization of DES-Based Hydrogels

#### 4.3.1. FTIR Spectroscopy

The dried DES gel samples were ground to a powder and thoroughly mixed with potassium bromide (KBr) powder at a mass ratio of 1:100 in an agate mortar (1 mg of gel powder and 100 mg of KBr). The mixture was then compressed into thin pellets using a tableting machine. Fourier transform infrared (FTIR) spectroscopy (Nicolet iS10, Thermo Fisher Scientific Inc., Madison, WI, USA) was used to characterize the prepared pellets. The spectra were recorded over a scanning range of 4000–400 cm^−1^ with a resolution of 4 cm^−1^ and 32 scans.

#### 4.3.2. Microstructure

The DES gel samples were immersed in deionized water until swelling equilibrium was reached, then freeze-dried in a vacuum freeze dryer for 24 h. The dried gels were frozen with liquid nitrogen and then fractured brittlely. The fractured cross-sections were sputter-coated with gold, and the micrographs were obtained using a scanning electron microscope (Sigma 300, ZEISS, Jena, Germany). Before image acquisition, all samples were fixed to the SEM stage with conductive adhesive, and a gold coating was applied to their surfaces using a sputtering coating machine. During imaging, an acceleration voltage of 3 kV was applied, and the beam current was maintained at 200–500 pA.

#### 4.3.3. Swelling Properties

After drying the DES gel samples, approximately 0.2 g of the dried gel was soaked in deionized water at room temperature (25 °C). When the gel reached swelling equilibrium through water absorption, it was removed, weighed, and recorded to analyze its swelling performance. All measurements were carried out in triplicate for statistical validation and error analysis. The swelling ratio (SR) was calculated using the following formula:

SR (%) = (Ws − Wd)/Wd × 100%

where Wd represents the dry weight of the hydrogel, Ws represents the wet weight of the hydrogel, and SR is the swelling ratio. The final results were presented as the mean ± standard deviation.

#### 4.3.4. Thermal Stability

The dried DES gel samples were ground into powder. Then, 10 mg of the gel powder was weighed and placed in a platinum pan of a thermogravimetric analyzer. Tests were carried out using a simultaneous thermal analyzer (STA8000, PerkinElmer, Shelton, CT, USA). Under a nitrogen atmosphere with a flow rate of 20 mL/min, the sample was heated from 30 °C to 800 °C at a heating rate of 10 °C/min.

#### 4.3.5. Mechanical Properties

The mechanical properties of the DES gels were tested using an INSTRON 5982 electronic universal testing machine (RGM-6300, INSTRON, Norwood, MA, USA, 1 kN). The gel samples were cut into cylinders with a diameter of 30 mm and a height of 15 mm. Compression tests were performed at a compression rate of 10 mm/min using a 1000 N load cell. All stress and strain data presented are true stress and strain. Each hydrogel sample was tested in triplicate, and the average value was used for the final result. All mechanical property tests were conducted under ambient conditions of 25 ± 2 °C and a relative humidity of 50 ± 5%.

## Figures and Tables

**Table 2:** Characteristic temperatures of thermogravimetric analysis (TGA) of hydrogels with different amounts of alkaline lignin additions.

Sample Code	T_5%_/(°C)	T_max1_/(°C)	R_max1_/(%·min^−1^)	T_max2_/(°C)	R_max2_/(%·min^−1^)	Residue at 700 °C/(%)
Gel L1-W2	100.64	260.71	−10.27	386.62	−3.57	9.02
Gel L2-W2	134.33	262.43	−10.43	386.77	−3.62	8.67
Gel L3-W2	219.37	270.59	−9.06	387.17	−3.77	8.55
Gel L4-W2	210.59	259.80	−9.97	385.16	−3.84	8.92
Gel L5-W2	120.38	261.21	−10.59	385.49	−3.51	8.98
Gel L6-W2	103.77	260.42	−11.52	387.44	−3.78	8.72

Note: T_5%_ represents the temperature at which gravity is reduced by 5%. T_max1,2_ represents the temperature corresponding to the maximum rate of weightlessness in the first and second stages. R_max1,2_ represents the maximum rate of weightlessness in the first and second stages.

**Table 3:** Characteristic temperatures of water gel under different water addition amounts during thermogravimetric analysis (TGA).

Sample Code	T_5%_/(°C)	T_max1_/(°C)	R_max1_/(%·min^−1^)	T_max2_/(°C)	R_max2_/(%·min^−1^)	Residue at 700 °C/(%)
Gel L2-W1	135.33	260.57	−11.62	390.34	−3.65	8.67
Gel L2-W2	125.67	261.44	−11.15	388.52	−3.56	8.54
Gel L2-W3	209.91	262.19	−9.88	390.11	−3.89	8.79

Note: T_5%_ represents the temperature at which gravity is reduced by 5%. T_max1,2_ represents the temperature corresponding to the maximum rate of weightlessness in the first and second stages. R_max1,2_ represents the maximum rate of weightlessness in the first and second stages.

## Data Availability

All data and materials are available on request from the corresponding author. The data are not publicly available due to ongoing research using a part of the data.
